# CD4^+^ T-Cells With High Common γ Chain Expression and Disturbed Cytokine Production Are Enriched in Children With Type-1 Diabetes

**DOI:** 10.3389/fimmu.2019.00820

**Published:** 2019-04-24

**Authors:** Julia Seyfarth, Nathalie Mütze, Jennifer Antony Cruz, Sebastian Kummer, Christina Reinauer, Ertan Mayatepek, Thomas Meissner, Marc Jacobsen

**Affiliations:** ^1^Department of General Pediatrics, Neonatology and Pediatric Cardiology, University Children's Hospital, Medical Faculty, Düsseldorf, Germany; ^2^German Center for Diabetes Research (DZD), Partner Düsseldorf, Düsseldorf, Germany

**Keywords:** biomarker, immunology, common gamma chain, interleukin-2, interleukin-7, type 1 diabetes, interleukin-15

## Abstract

The common gamma chain (γ_c_) contributes to the formation of different cytokine receptors [e.g., IL-2 receptor (IL-2R), IL-7R, and IL-15R], which are important for generation of self-reactive T-cells in autoimmune diseases, like in type 1 diabetes (T1D). Whereas, the roles of membrane and soluble IL-2Rα and IL-7Rα variants in T1D disease pathogenesis are well-described, effects of γ_c_ expression and availability for dependent receptors remain elusive. We investigated expression of the γ_c_ and dependent receptors on T-cells and soluble γ_c_ concentrations in serum from patients with T1D (*n* = 34) and healthy controls (*n* = 27). Effector T-cell cytokines as well as IL-2, IL-7, and IL-15 induced STAT5 phosphorylation were analyzed to determine functional implications of differential γ_c_ expression of CD4^+^ T-cell subsets classified by t-distributed Stochastic Neighbor Embedding (t-SNE) analyses. We found increased γ_c_ and IL-7Rα expression of CD4^+^ T-cells from T1D patients as compared to controls. t-SNE analyses assigned differential expression to subsets of memory T-cells co-expressing γ_c_ and IL-7Rα. Whereas, γ_c_ expression was positively correlated with IL-2Rα in memory T-cells from healthy controls, no dependency was found for patients with T1D. Similarly, the effector T-cell cytokine, IL-21, correlated inversely with γ_c_ expression in healthy controls, but not in T1D patients. Finally, T1D patients with high γ_c_ expression had increased proportions of IL-2 sensitive pSTAT5^+^ effector T-cells. These results indicated aberrantly high γ_c_ expression of T-cells from T1D patients with implications on dependent cytokine receptor signaling and effector T-cell cytokine production.

## Introduction

Type 1 diabetes (T1D) is an autoimmune disease characterized by destruction of pancreatic beta islet cells. Self-reactive effector T-cells are found in T1D and contribute to disease pathogenesis. Mainly T helper type (T_H_) 1 cells, producing the key cytokines IFN-γ and TNF-α, were detected in T1D-specific pancreas islet inflammation (1), but also other CD4^+^ T-cell subsets, e.g., producing IL-17 or IL-21, have been found at higher frequencies in peripheral blood from patients with T1D (2, 3). IL-21 is a key cytokine of T follicular helper (T_FH_) cells, which are central for B-cell support and may play a role for generation of auto-antibodies in T1D (2).

The T-cell repertoire of patients with T1D is generally prone to increased basic activation (4, 5). Possible explanations for this are increased regulation resistance of effector T-cells and/or impaired regulatory T-cell (T_reg_) functions (6, 7). Members of the γ_c_ cytokine family, namely IL-2, IL-7, and IL-15 are crucially involved in T-cell regulation and the generation of effector T-cells. IL-2 and IL-7 exert largely contrary roles with IL-2 promoting T_reg_ cells and IL-7 being essential for generation of effector and memory T-cells (8). Especially the generation of self-reactive T-cells depends on IL-7 potentially by lowering the T-cell activation threshold (9). IL-15 is an IL-7 related cytokine with similar functions for CD8^+^ T-cells and NK cells and potential relevance for autoimmunity (10).

Membrane IL-2Rα and IL-7Rα expression of T-cells affect their response to respective cytokines and both receptors are strongly regulated during T-cell activation and maturation (8). The relevance of IL-2Rα and IL-7Rα regulation for development of autoimmune diseases including T1D has been clearly shown (11–13). Both, IL-2R and IL-7R, are members of the γ_c_ cytokine receptor family, characterized by γ_c_ chain dependency for receptor formation and cytokine signaling. Only limited evidence for regulation of γ_c_ expression has been found so far. The majority of previous studies reported stable γ_c_ expression in T-cells and assumed that differences in γ_c_ availability are functionally irrelevant (14). However, there is some evidence for γ_c_ upregulation during T-cell activation and differential γ_c_ expression in T-cell subpopulations (15, 16). In addition, modified availability of γ_c_, e.g., due to occupation of individual receptor chains, has been assumed to affect the response against γ_c_ cytokines (17, 18). In this regard, observations from patients with γ_c_ gene deficiency demonstrated that IL-7, IL-2 and IL-15 require different levels of γ_c_ for optimal signaling (19, 20). In support of these findings, Monti et al. showed that disengagement of the IL-2Rα chain with daclizumab enhanced T-cell responses for IL-7 and demonstrated the functional relevance of γ_c_ availability (21).

Evidence for a potential role of γ_c_ in T1D pathology comes from the study of Demirci et al. who showed that antibodies against γ_c_ prevented T1D disease onset in animal models (22). Chronic inflammatory diseases may also be affected by γ_c_ expression since increased serum concentrations of the soluble (s)γ_c_ variant were reported in human inflammatory bowel disease (23) and rheumatoid arthritis (24, 25).

In the present study, we compared expression of γ_c_ cytokine receptor chains between patients with T1D and matched healthy controls. Associations between individual chain expression and phenotype of different T-cell subpopulations were examined by t-distributed Stochastic Neighbor Embedding (t-SNE) analyses. Finally, T-cell activation induced intracellular cytokine expression pattern and cytokine induced STAT5 phosphorylation were characterized.

## Results

### γ_c_ and IL-7Rα Expression Levels Are Higher in CD4^+^ Memory T-Cells From T1D Patients as Compared to Healthy Controls

**Figure 1:**
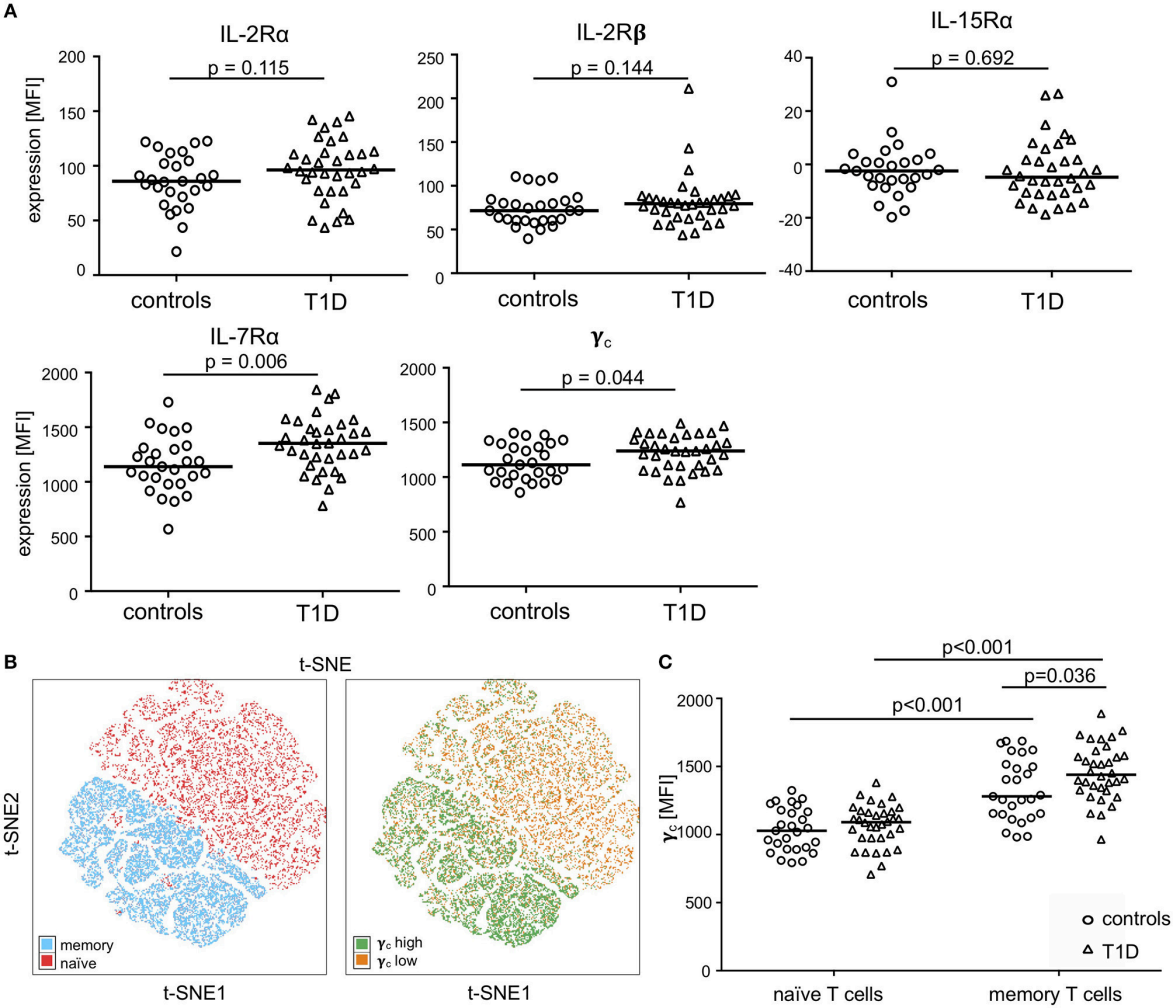
Expression of the γ_c_ cytokine receptor chains on naïve and memory CD4^+^ T-cells. **(A)** Expression of γ_c_, IL-7Rα, IL-2Rα, IL-2Rß and IL-15Rα on CD4^+^ T-cells of healthy children (controls, *n* = 27, open circles) and children with T1D (T1D, *n* = 34, open triangles) are shown as (geometric) mean fluorescence intensity (MFI). Each symbol represents the mean of triplicates for an individual donor. Median values of groups are indicated and nominal *p*-values of the two-tailed Mann-Whitney U-test are provided. **(B)** Unbiased t-distributed Stochastic Neighbor Embedding (t-SNE) analysis of a concatenated subgroup of CD4^+^ T-cells from healthy controls (*n* = 11) and T1D patients (*n* = 19) illustrate distribution of naïve CD45RA_high_ and memory CD45RA_low_ (red and blue, respectively; left panel) and γ_c_ high (top 10% mean fluorescence of all CD4^+^ cells; green) and γ_c_ low (bottom 90% mean fluorescence; orange) (right panel) CD4^+^ T-cells. t-SNE calculates two-dimensional depiction of multi-factorial similarity. These two dimensions are characterized by t-SNE1 and t-SNE2 in given graphs. **(C)** γ_c_ expression of naïve CD45RA_high_ and memory CD45RA_low_ CD4^+^ T-cells are shown for healthy controls (*n* = 27, open circles) and T1D patients (*n* = 34, open triangles). Median values of groups and statistically significant nominal *p*-values for the Mann-Whitney U-test (two-tailed) are indicated.

We determined expression of IL-2R, IL-7R, and IL-15R chains on CD4^+^ T-cells from children with T1D (*n* = 34) as well as healthy controls (“controls”; *n* = 27). Donor characteristics are summarized in Table 1. No differences in mean expression were detected for the IL-2Rα, the IL-2Rβ, and the IL-15Rα chain between the study groups (Figure 1A, upper graphs; for gating strategy see Supplementary Figure 1A). Interestingly, children with T1D had higher mean expression of IL-7Rα (*p* = 0.006) and γ_c_ (*p* = 0.044) on CD4^+^ T-cells as compared to healthy controls (Figure 1A, bottom graphs). To further characterize affected T-cell subsets, we applied the unbiased approach of t-distributed Stochastic Neighbor Embedding (t-SNE) analysis for two-dimensional visualization of high-dimensional data (26). Figure 1B shows combined flowcytometry data of CD4^+^ T-cells from T1D patients and controls (for gating strategy see Supplementary Figures 1A,B). Naïve and memory T-cells were classified by CD45RA_high_ and CD45RA_low_ expression, respectively (Figure 1B, left graph). γ_c_ high T-cells (top 10% according to mean γ_c_ expression) clustered almost exclusively within the memory CD4^+^ T-cell subset (Figure 1B, right graph). This suggested higher γ_c_ expression in memory CD4^+^ T-cells. Hence, we next compared γ_c_ expression between naïve and memory T-cells from both study groups. As expected, γ_c_ expression was generally higher in memory T-cells as compared to naïve T-cells (Figure 1C, *p* < 0.001, for T1D patients and controls). Study group comparisons revealed that higher γ_c_ expression was exclusively detected for memory T-cells of T1D patients (*p* = 0.036).

**Table 1:** Baseline characteristics of children with T1D and healthy controls.

**Characteristic**	**Healthy controls**	**T1D patients**	***p*-Value**
Number (*n*)	27	34	
Age (years)	13.0 [3.5–17.5]	13.9 [4.4–17.7]	0.888
Sex distribution (m/f)	15/12	20/14	0.798
Disease duration	na	5.5 years [0.9–14.7 years]	
HbA1c (%)	nd	7.7 [6.4–9.6]	
HbA1c (mmol mol^−1^)	nd	61 [46–81]	
C-peptide (μg l^−1^)	nd	< 0.01 [ < 0.01–1.96]	

*f, female; m, male; na, not applicable; nd, not defined. Median and [range] are given. P-values were based on the Mann-Whitney U-test (for the continuous variable age) and chi-squared test (for the categorical variable sex distribution)*.

### Identification of a γ_c_ and IL-7Rα High Expressing CD4^+^ Memory T-Cell Subset Enriched in T1D Patients

**Figure 2:**
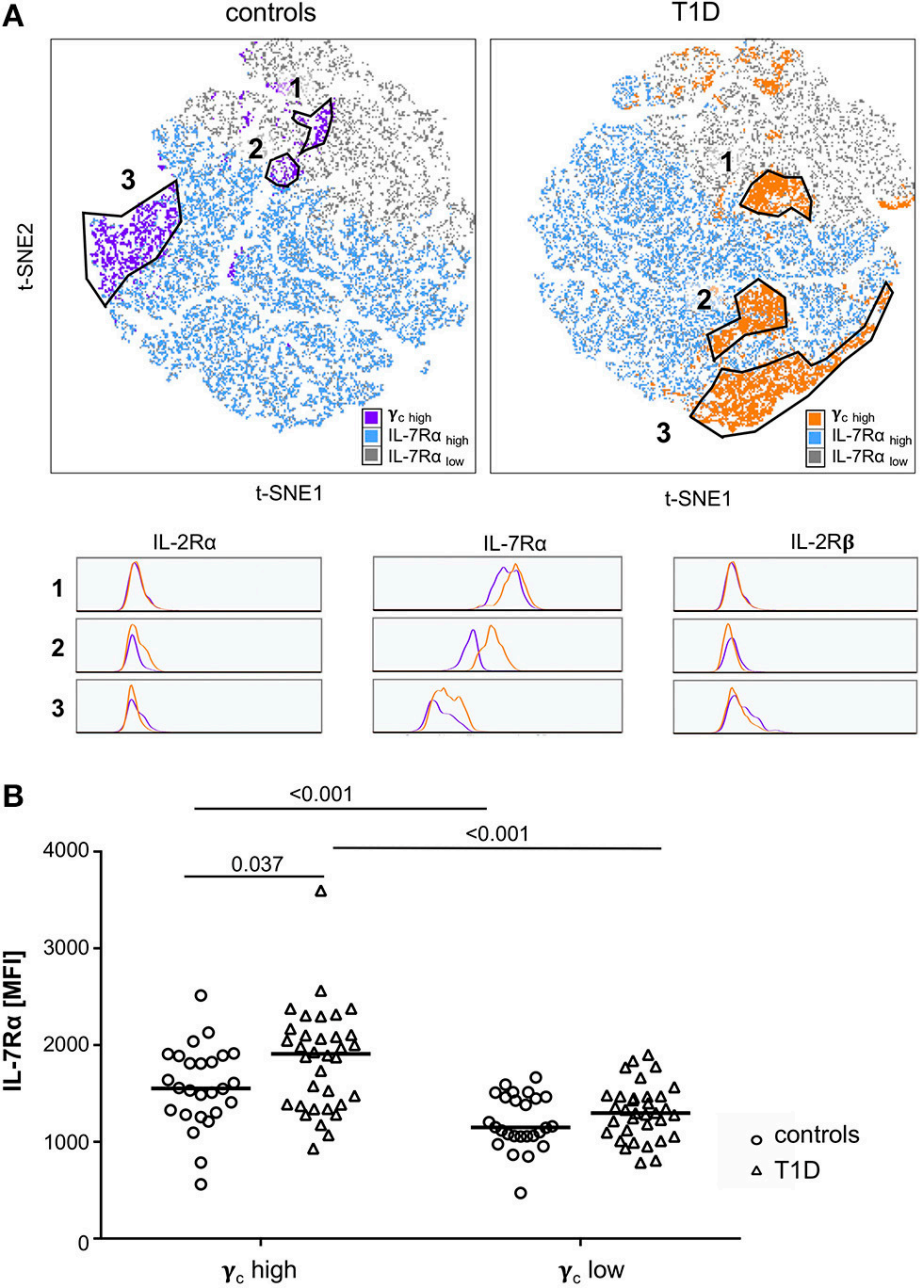
Characterization of γ_c_ high expressing memory T-cell populations. **(A)** Unbiased t-distributed Stochastic Neighbor Embedding (t-SNE) analysis of memory CD4^+^ T-cells (i.e., CD45RA_low_) from healthy controls (*n* = 20, left graph) and T1D patients (*n* = 25, right graph). IL-7Rα high cells (blue), IL-7Rα low cells (gray), and γ_c_ high cells (purple for controls; orange for T1D patients) are illustrated. γ_c_ high populations (top10% mean fluorescence of all CD4^+^/CD45RA_low_ cells) of controls and patients were gated (populations 1, 2, and 3) and compared for the respective IL-2Rα, IL-7Rα and IL-2Rβ expression (histograms). **(B)** IL-7Rα expression of γ_c_ high and γ_c_ low cells is shown for healthy controls (*n* = 27, open circles) and T1D patients (*n* = 34, open triangles). Median values of groups and statistically significant nominal *p*-values for the Mann-Whitney U-test (two-tailed) are indicated.

To further characterize γ_c_ high memory T-cell subsets and to compare study groups, we performed t-SNE analyses for subgroups of patients with T1D and controls separately. Three main populations were identified with high γ_c_ expression (top 10% according to mean γ_c_ expression; for gating strategy see Supplementary Figure 1B) within CD4^+^ memory T-cells for both study groups (Figure 2A, t-SNE plots). Higher similarity, indicated by distance in t-SNE principal component analyses, was suggested for high γ_c_ expressing subpopulations 1 and 2 in controls whereas subpopulations 2 and 3 were more similar in T1D patients (Figure 2A). In accordance, subpopulation 2 clustered in a region of IL-7Rα high expressing memory T-cells from patients (Figure 2A, upper right plot) whereas lower IL-7Rα expression characterized subpopulation 2 in healthy controls (Figure 2A, upper left plot; for gating strategy see Supplementary Figure 1B). γ_c_ high subpopulations showed comparable expression of IL-2Rα and IL-2Rβ between both study groups (Figure 2A; histograms), whereas higher IL-7Rα mean expression of γ_c_ high memory T-cells—especially for subpopulation 2—was detected for T1D patients (Figure 2A; histograms). Hence, we compared γ_c_ high and low T-cells for IL-7Rα expression between patients and controls. γ_c_ low T-cells expressed generally less IL-7Rα in both study groups as compared to γ_c_ high T-cells (*p* < 0.001 for patients and controls) and no differences were found for γ_c_ low T-cells between study groups (Figure 2B). In contrast γ_c_ high T-cells from patients with T1D expressed significantly higher IL-7Rα levels as compared to healthy controls (*p* = 0.037; Figure 2B). These results indicated that γ_c_/IL-7Rα high co-expressing T-cell proportions were enriched in T1D patients.

### Loss of Correlation of γ_c_ With IL-2Rα on Memory CD4^+^ T-Cells From Patients With T1D

**Figure 3:**
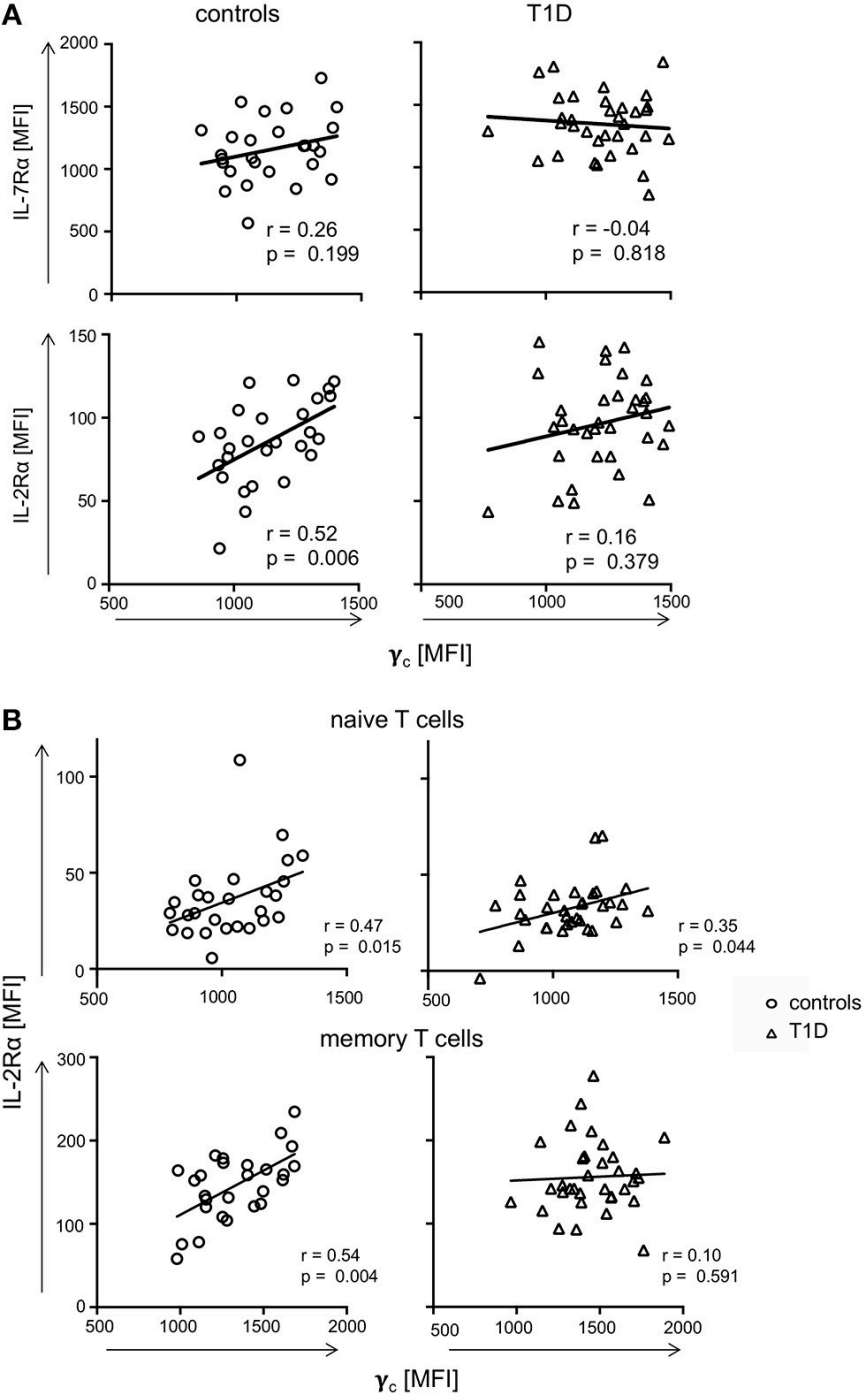
Correlations of γ_c_ with IL-7Rα and IL-2Rα. **(A)** Correlations of IL-7Rα (upper panels) and IL-2Rα (lower panels) with γ_c_ on CD4^+^ T-cells are shown for healthy controls (left graphs, *n* = 27, open circles) and T1D patients (right graphs, *n* = 34, open triangles). **(B)** Correlations of IL-2Rα with γ_c_ on naïve (i.e., CD45RA_high_ CCR7_high_) (upper panels) and memory CD45RA_low_ (lower panels) CD4^+^ T-cells are shown for healthy controls (left panels, *n* = 27, open circles) and T1D patients (right panels, *n* = 34, open triangles). **(A,B)** Spearman rank correlation coefficients *r* and *p*-values are indicated. A trend line was fitted by linear regression analysis.

Availability of γ_c_ has been shown to affect T-cell cytokine signaling for γ_c_ family members (19, 21). Therefore, we next measured relative expression of γ_c_ cytokine receptor chains in individual donors. γ_c_ did not show significant correlation with IL-7Rα expression for CD4^+^ T-cells from controls or patients with T1D (Figure 3A, upper graphs). Similar results were gained for the IL-2Rβ chain (Supplementary Figure 2). In contrast, γ_c_ correlated positively with IL-2Rα chain expression for healthy controls (*r* = 0.52, *p* = 0.006), whereas no correlation between γ_c_ and IL-2Rα was detectable for patients (*r* = 0.16, *p* = 0.379) (Figure 3A, lower graphs). Since differential γ_c_ expression was only found for memory T-cells, we next compared γ_c_ and IL-2Rα on CD4^+^ naïve and memory subsets. We found significant correlation between γ_c_ and IL-2Rα for both naïve and memory T-cells of healthy controls (*r* = 0.47, *p* = 0.015; *r* = 0.54, *p* = 0.004, respectively) (Figure 3B, left graphs). In patients, however, only naive T-cells showed a moderate correlation (*r* = 0.35, *p* = 0.044) whereas no correlation was detectable for memory CD4^+^ T-cells (*r* = 0.10, *p* = 0.591) (Figure 3B, right graphs). We concluded that differential γ_c_ expression of patients with T1D abrogated positive correlation with IL-2Rα on memory T-cells found in healthy controls.

### Negative Correlation of γ_c_ With Cytokine Expression of CD4^+^ Memory T-Cells Is Absent in Patients With T1D

**Figure 4:**
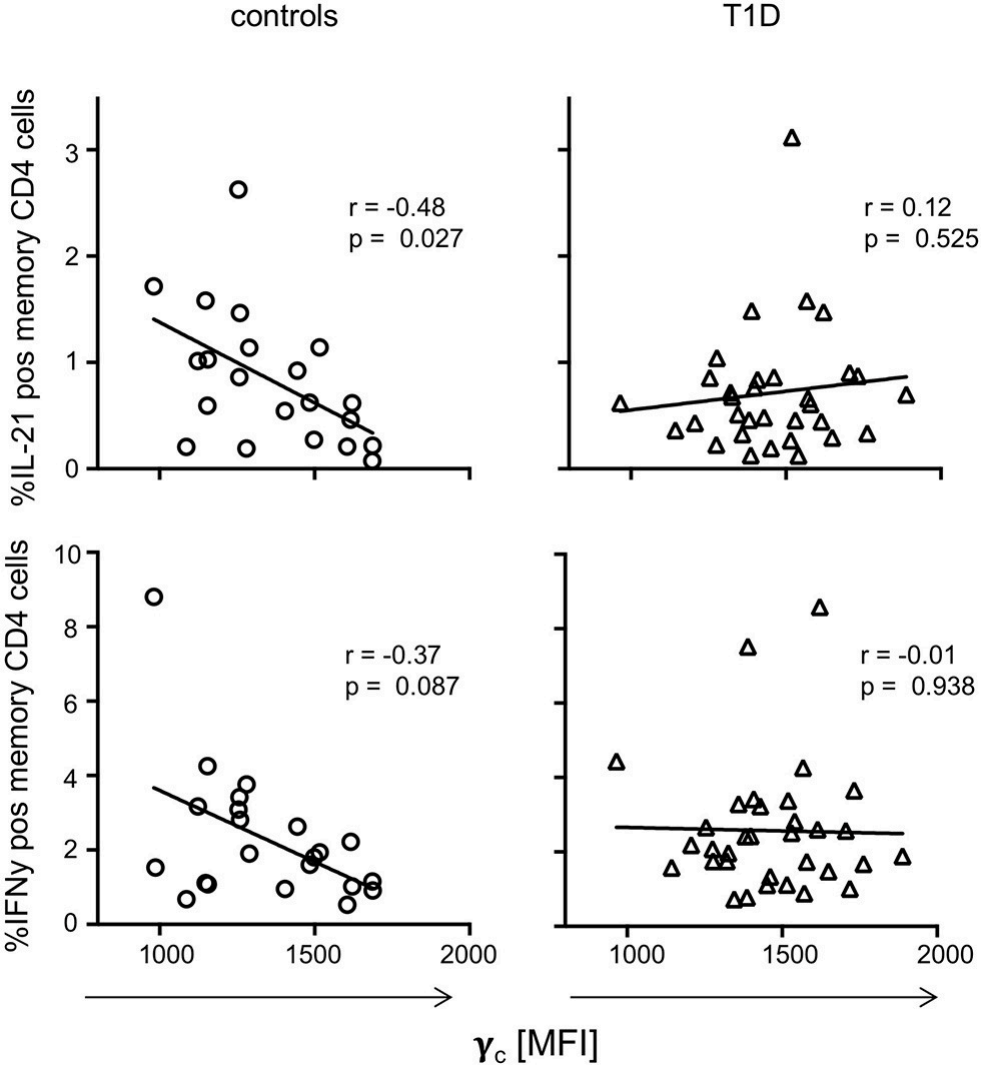
γ_c_ expression and cytokine production on T cell receptor stimulation. Correlation between γ_c_ expression on CD4^+^ memory T-cells and *in vitro* activated cytokine expressing memory CD4^+^ T-cells (upper graphs: IL-21, bottom graphs: IFNy) are shown for healthy controls (*n* = 21, left graphs) and patients with T1D (*n* = 33, right graphs). Spearman rank correlation coefficients (*r*) and respective *p*-values are indicated. A trend line was fitted by linear regression analysis.

To evaluate if dysregulated γ_c_ expression in memory T-cells from T1D patients affects CD4^+^ T-cell function, we next assessed *in vitro* T-cell activation induced cytokine production for T1D patients and controls. IFNγ, IL-21, TNFα, and IL-2 expressing CD4^+^ memory T-cell proportions (see Supplementary Figure 3A for gating procedures) were compared with individual γ_c_ expression on memory CD4^+^ T-cells. For healthy controls, we detected negative correlation of γ_c_ expression with IL-21 producing CD4^+^ memory T-cells (*r* = −0.48, *p* = 0.027) and a tendency for IFNγ (*r* = −0.37, *p* = 0.087) (Figure 4, left panel). In contrast, patients showed no correlation of γ_c_ expression with IL-21 or IFNγ producing CD4^+^ memory T-cells (Figure 4, right panel). No correlations were found for γ_c_ expression and TNFα/IL-2 expression, neither for controls nor for T1D patients (Supplementary Figure 3B). This suggested that the high γ_c_ expression in T1D abrogated the negative association between γ_c_ expression of CD4^+^ memory T-cells and IL-21 production found in controls.

### Type 1 Diabetes Patients With High γ_c_ Expression on CD4^+^ T-Cells Have Increased Proportions of IL-2 Induced pSTAT5 Positive T-Cells

**Figure 5:**
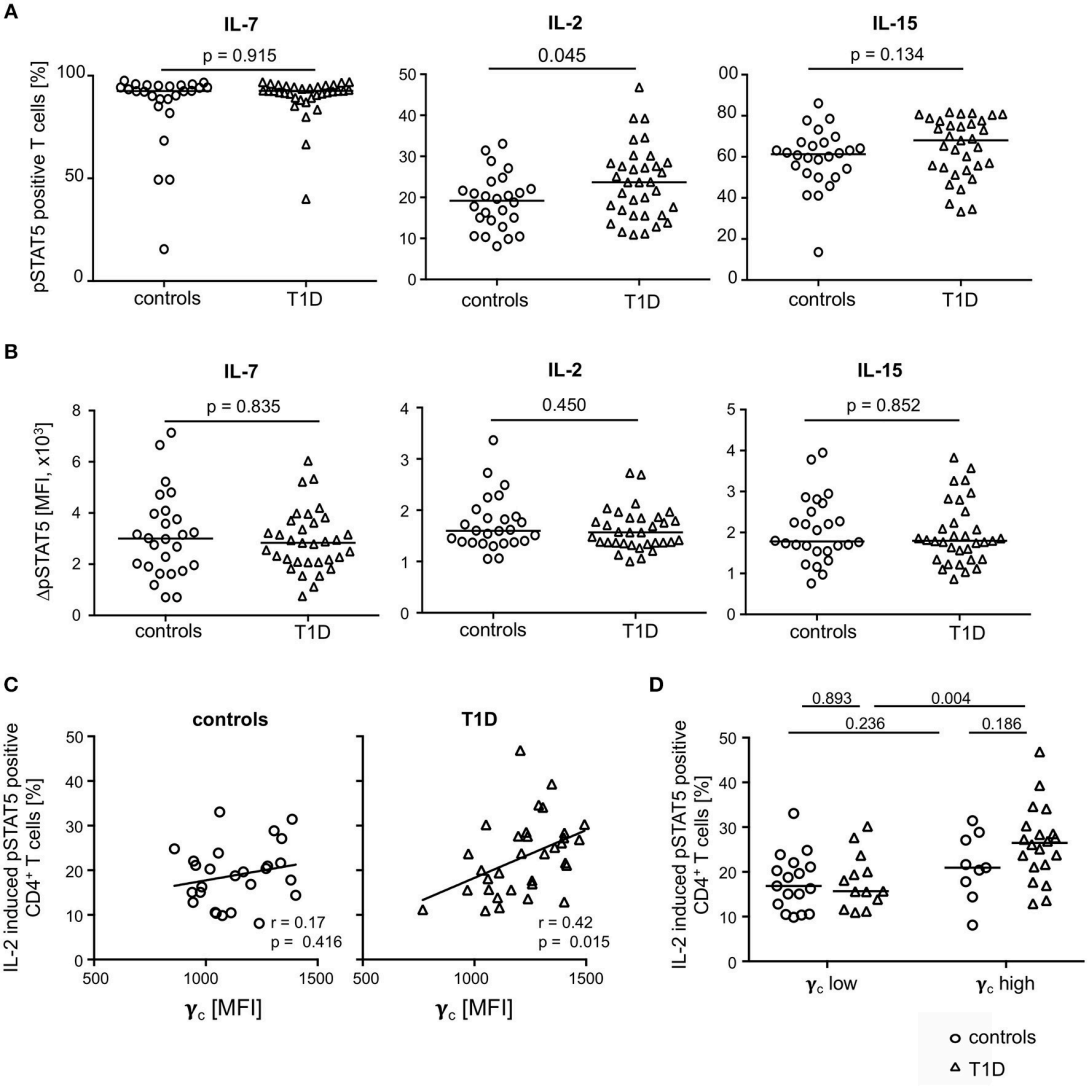
Cytokine induced STAT5 phosphorylation in CD4^+^ T-cells from healthy controls and patients with T1D. Proportions of pSTAT5 positive CD4^+^ T-cells **(A)** and ΔpSTAT5 mean fluorescence intensity (MFI) of CD4^+^ T-cells **(B)** induced by IL-7 (left graph), IL-2 (middle graph) and IL-15 (right graph) are shown for healthy controls (*n* = 26) and T1D patients (*n* = 34). ΔpSTAT5 MFI was calculated as follows: MFI (pSTAT5 positive cells)—MFI (unstimulated cells). Every symbol represents the mean of duplicates measured for an individual donor. Median values of groups and nominal *p*-values for the Mann-Whitney U-test (two-tailed) are indicated. **(C)** Correlations between γ_c_ expression on CD4^+^ T-cells and proportions of IL-2 induced pSTAT5 positive CD4^+^ T-cells are shown for controls (left panel) and children with T1D (right panel). Spearman rank correlation coefficients (*r*) and respective *p*-values are indicated. A trend line was fitted by linear regression analysis. **(D)** Study participants were stratified into a “γ_c_ low” and a “γ_c_ high” subgroup according to γ_c_ expression (below or above the median γ_c_ MFI expression) on CD4^+^ T-cells. Proportions of pSTAT5 positive CD4^+^ T-cells were compared between healthy controls and T1D and between both subgroups. Median values of groups and nominal *p*-values for the Mann-Whitney U-test (two-tailed) are indicated.

To address the question of functional impacts on cytokine signaling, we measured IL-2, IL-7, and IL-15 induced STAT5 phosphorylation of CD4^+^ T-cells from both study groups. The gating procedure of cytokine induced pSTAT5 expression is depicted in Supplementary Figure 4A. IL-7 induced pSTAT5 in the vast majority of CD4^+^ T-cells (median: 92.6%) and no differences between the study groups were detected (Figure 5A, left graph). In contrast, IL-2 increased the proportion of pSTAT5 positive T-cells in T1D patients as compared to healthy controls (Figure 5, middle graph; *p* = 0.045) whereas proportions were not significantly different after IL-15 stimulation (Figure 5A, right graph). Interestingly, mean pSTAT5 expression of positive T-cells was similar between the study groups independent of the respective cytokine (Figure 5B). This suggested that patients with T1D have more IL-2 sensitive CD4^+^ T-cells than controls but no differences in the IL-2 induced signaling intensity as compared to control T-cells.

Procedures used for measurement of phosphorylated proteins by flow cytometry rendered concomitant cytokine receptor analyses not feasible. However, to identify potential effects of differential γ_c_ expression between the study groups on IL-2 induced signaling, we correlated both in patients with T1D patients and controls. Whereas, no correlation of γ_c_ expression and IL-2 induced signaling was found for healthy controls (Figure 5C; left graph), a significant positive correlation was found for γ_c_ expression and IL-2 induced pSTAT5 proportions for patients (*r* = 0.42; *p* = 0.015; Figure 5C, right graph).

These findings may be caused by different proportions of γ_c_ low or high T-cells. To address this question, we classified T1D patients and healthy controls as γ_c_ low or high (for details see Methods) and compared subgroups for pSTAT5 positive T-cell proportions. Comparisons of IL-2 induced pSTAT5 positive T-cells showed increased proportions in CD4^+^ T-cells from γ_c_ high as compared to γ_c_ low T1D patients (*p* = 0.004) (Figure 5D) and a similar tendency was seen for IL-15 (*p* = 0.068) (Supplementary Figure 4B, right graph). Notably, no differences were seen when comparing IL-2/IL-15 induced γ_c_ high and γ_c_ low healthy controls (Figure 5D). IL-7 induced pSTAT5 proportions were also similar between γ_c_ high and low individuals as well as between both study groups (Supplementary Figure 4B, left graph). Differences between the study groups were not due to differential mean γ_c_ since γ_c_ high and γ_c_ low from the study groups had comparable values (Supplementary Figure 5A). Furthermore, regulatory T (T_reg_) cells were likely not causative for differential IL-2/IL-15 response since T_reg_ proportions (for gating strategy see Supplementary Figure 5B) were similar between γ_c_ high and γ_c_ low subgroups (Supplementary Figure 5B). These results argued for an increased number of CD4^+^ T-cells with increased sensitivity for IL-2 (partly IL-15) in type 1 diabetes patients with higher γ_c_ expression.

### No Differences of Soluble γ_c_ Serum Levels Between T1D Patients and Controls and No Correlation With Membrane-Associated γ_c_ Expression

**Figure 6:**
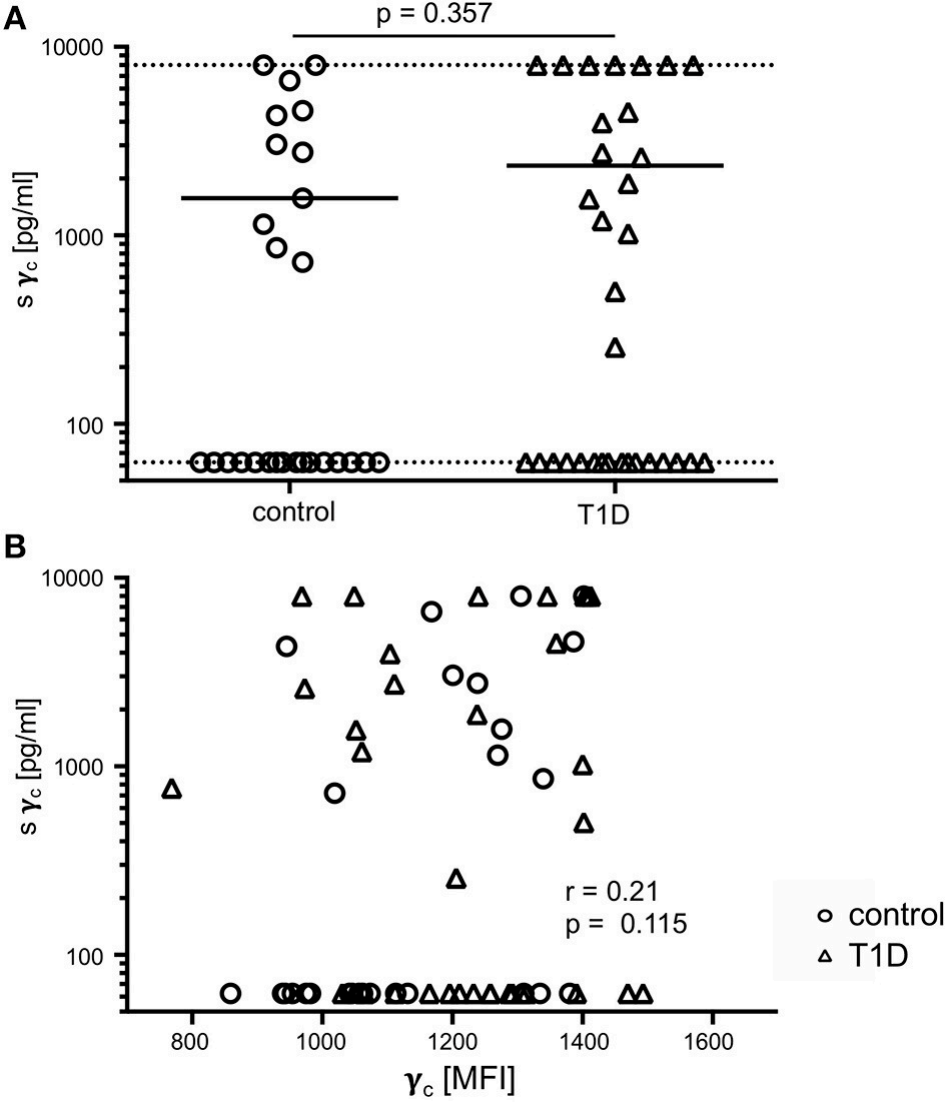
Soluble (s) and membrane-associated γ_c_ expression. **(A)** Serum concentrations of sγ_c_ measured by ELISA are shown for healthy controls (*n* = 27, open circles) and T1D patients (*n* = 33, open triangles). The dotted lines indicate the upper and lower detection limit. Every symbol represents the mean of duplicates measured for an individual donor. Mean values of groups and the nominal *p*-value for the Mann-Whitney U-test (two-tailed) are indicated. **(B)** Correlation of serum concentrations of sγ_c_ with membrane-associated γ_c_ on CD4^+^ T-cells are shown (open circles: healthy controls, open triangles: patients with T1D). Spearman rank correlation coefficient (*r*) and *p*-value are indicated.

In previous studies, soluble γ_c_ levels in serum were shown to be affected in autoimmune pathologies (23, 24). Hence, we compared soluble γ_c_ concentrations in serum from T1D patients and healthy controls. Soluble γ_c_ concentrations differed strongly between individuals and the vast majority of individuals from both study groups (16/27 = 59.3%, 16/33 = 48.5% of patients and controls, respectively) had no detectable soluble γ_c_ concentrations in serum (Figure 6A). No significant differences between study groups were found (*p* = 0.357) (Figure 6A). Finally, membranous γ_c_ expression was not correlated with soluble γ_c_ concentrations (*r* = 0.21, *p* = 0.115) (Figure 6B). These results indicated no association between membranous γ_c_ expression and soluble γ_c_ serum levels.

## Discussion

T1D-specific differences in cytokine expression and activation pattern of memory T-cell populations have been described and a role of γ_c_ cytokines IL-2 as well as IL-7 is likely. T-cell sensitivity for IL-2 and IL-7 depends on IL-2Rα and IL-7Rα receptor expression levels (8). Furthermore, initial studies indicated a role of the shared γ_c_ receptor and its soluble variant in autoimmune pathogenesis (22, 25, 27). Therefore, we performed a case-control study and identified higher γ_c_ and IL-7Rα chain expression on CD4^+^ T-cells from T1D patients. Notably, γ_c_ expression of memory CD4^+^ T-cells from patients with T1D was accompanied by the absence of positive correlation with the IL-2Rα found for healthy controls. Since this positive correlation was preserved in naïve T-cells from patients with T1D, we concluded that T1D-specific changes of γ_c_ expression occur during effector and memory T-cell generation. The absence of correlation between γ_c_ and IL-2Rα together with generally increased γ_c_ expression suggested T1D-specific γ_c_ up-regulation in IL-2Rα low or medium T-cells. In accordance, t-SNE analysis pointed toward γ_c_ up-regulation in memory T-cells with moderate IL-2Rα expression and concomitantly high IL-7Rα and γ_c_ expression of memory T-cells from T1D patients.

So far, only few studies focused on γ_c_ expression of T-cells in autoimmune and inflammatory diseases. This may be explained by the prevailing assumption that γ_c_ expression is constitutive and not transcriptionally regulated (14). However, several studies report upregulation of γ_c_ expression after cytokine and T-cell receptor activation (15, 16, 27) or during infections (16, 28). Mechanistically, γ_c_ was shown to be stored intracellularly and to be translocated to the plasma membrane after T-cell activation (16, 29). Causative mechanisms underlying higher γ_c_ expression of memory T-cells from patients with T1D could not be addressed in the present study since cytokine receptor chains were only measured on the plasma membrane. Comparison of intracellular γ_c_ expression levels as well as on the mRNA level would be needed to reveal potential causes.

To elucidate the impact of increased γ_c_ expression on T-cell function, we correlated γ_c_ expression with memory CD4^+^ T-cell cytokine production in T1D patients and healthy controls. We found a negative association of γ_c_ expression with IL-21 production exclusively in healthy controls whereas no correlation was found for children with T1D. Although not significant, similar results were gained for IFN-γ positive T-cells. These results suggested that, under “healthy” conditions, high γ_c_ expression levels are found in donors with low IL-21 expression, indicating negative effects of γ_c_ high expressing cells on IL-21 cytokine production. Abrogation of γ_c_ negative correlation could indicate that γ_c_ high IL7Rα co-expressing T-cells produce IL-21 or promote IL-21 producing T-cells in T1D patients. Accordingly, previous studies suggested that IL-21 significantly contributes to T1D development (30). Direct proof for IL-21 promoting effects of γ_c_ high T-cells from T1D patients would have required FACS-based sorting of γ_c_ high and low T-cell subsets from children with T1D and healthy controls. This approach and additional experiments to elucidate underlying mechanisms were not feasible because of the limited blood sample volume available from participating children.

Indication of a potential functional impact of differential γ_c_ expression is provided by pSTAT5 analyses and identification of higher IL-2 (partly IL-15) sensitivity in a subset of CD4^+^ T-cells from T1D patients characterized by high γ_c_ expression. Own previous studies had indicated higher IL-7 sensitivity of effector memory T-cells from T1D patients leading to increased T-cell activation in the presence of IL-7 (31). Hence, we assumed that enhanced IL-7 mediated T-cell responses in T1D patients could be due to higher γ_c_ levels. However, no differences in IL-7 induced STAT5 phosphorylation were found between the study groups and similar pSTAT5 levels were seen for subgroups classified for differential γ_c_ within T1D patients and controls. Although IL-7Rα was increased in T cells from T1D patients, these results did not suggest an association between γ_c_ expression and IL-7 mediated T-cell responses. In contrast, we detected increased IL-2 (and a tendency for IL-15) sensitive γ_c_ high CD4^+^ T-cell proportions in T1D patients. Although direct association analyses were not possible in STAT5 phosphorylation assays, we concluded that cytokine sensitivity of γ_c_ high T-cell proportions promoted IL-2 (IL-15) rather than IL-7. Evidence for a role of differential γ_c_-dependent γ_c_ cytokine signaling come from several animal models including experimental autoimmune encephalitis (EAE) and rheumatoid arthritis (25, 27, 32–34). Hong et al. assessed implications of the sγ_c_ chain on cytokine signaling and found that this inhibitory variant especially blocked IL-2R signaling probably by binding to the IL-2Rß chain and preventing the association with membrane γ_c_ proteins (27). Sγ_c_ mediated impaired IL-2 response of T cells caused increased IL-17 production and worsening of EAE and arthritis symptoms (25, 27). Here we detected a promoted IL-2 response in T1D patients with high membrane γ_c_ expression. Since Th17 differentiation is negatively regulated by IL-2 signaling (35), future studies will address the question if IL-17 expression is impaired in high γ_c_ expressing T cells. This may contribute to the recent assumption that Th17 cells may be increased, but also decreased in the context of T1D (36).

Previous studies focused on limiting membrane γ_c_ expression levels and competition of γ_c_ family cytokines under such circumstances. Smyth et al. showed, that under conditions where the availability of γ_c_ is limiting (in patients with x-SCID and trace amounts of correctly spliced γ_c_), IL-2 and IL-15 stimulation was preserved. In contrast, IL-7 stimulation did not induce pSTAT5, suggesting that IL-2/IL-15 signaling needs less γ_c_ expression as compared to IL-7R-mediated signaling (19). Hierarchies of cytokine responses may be due to differential capacity to bind γ_c_ and thereby reduce γ_c_ availability for other family members (18). Future studies are necessary to address the question, how increased γ_c_ expression differentially favors γ_c_ cytokine signaling.

Since previous reports indicated a role of soluble γ_c_ in autoimmunity, we measured soluble γ_c_ concentrations in the serum of patients with T1D and healthy controls. In accordance with previous studies, marked variability in soluble γ_c_ serum concentrations was found (23), however, no differences between T1D patients and controls were detected.

In summary, our study suggests a potential role of membranous γ_c_ expression in memory T-cells for T1D pathophysiological mechanisms. Future studies will have to shed light on the question, how dysregulated γ_c_ expression is involved in T1D development and maintenance.

## Methods

### Donor Characteristics

We recruited children and adolescents with T1D (*n* = 34) and healthy controls (*n* = 27) at the University Children‘s Hospital, Duesseldorf, Germany. Children with T1D had clinical manifestation more than 11 months ago. The control group consisted of children with negative history for autoimmune and systemic inflammatory diseases. Study group characteristics are given in Table 1.

### Surface Staining of γ_c_ Receptor Chains

For *ex vivo* surface staining, 100 μl of blood was immediately diluted in equal parts with DPBS. After centrifugation, the cell pellet was stained with an antibody mixture containing the following antibodies: For identification of T helper cells, we included CD4-BV510 (OKT4, Biolegend) and CD8-BV785 (RPA-T8, Biolegend). For identification of naïve, central and effector memory T-cells, we used CD45RA-FITC (HI100, Biolegend) and CCR7-PE-Cy7 (3D12, BD). In addition, the γ_c_ cytokine receptors IL-7Rα AF700 (clone A019D5, Biolegend), IL-2Rα-PerCP/Cy5.5 (BC96, Biolegend), IL-2Rβ (CD122)-PE (TU27, Biolegend), IL-15Rα (CD215)-APC (JM7A4, Biolegend), and γ_c_ (CD132)-PE-CF594 (TUGh4, BD) were included. Fixable viability dye eFluor780 (Thermo Fisher Scientific) was used to exclude dead cells. Staining was performed in triplicates. Sample measurement was performed on a LSR Fortessa flow cytometer (BD Biosciences). For data analysis FlowJo software (Miltenyi Biotech) was used. The gating procedure is depicted as Supplementary Figures 1A,B. Dead cells (viability marker positive cells) were excluded.

### t-SNE Analysis

t-distributed Stochastic Neighbor Embedding (t-SNE) analysis (26) was done by using a plugin in FlowJo v10.4. t-SNE calculations were performed with 1,000 iterations, a perplexity of 20, an Eta (learning rate) of 200 and a Theta of 0.5. t-SNE visualizes similarities of cells in a 2D plot, illustrating their proximity by their distances in the t-SNE map. This method has been previously used to visualize different cellular subpopulations (24). In other words, each dot in the t-SNE plot represents a cell from an individual study participant and the distance between two dots/cells indicates their similarity. E.g., each T-cell population marked in Figure 2A because of high γ_c_ expression contains memory CD4^+^ T-cells with high similarity. In contrast, different populations (i.e., 1, 2, and 3) share features (here high γ_c_ expression) but are different in other parameters (here IL-7Rα) expression.

t-SNE calculates two-dimensional depiction of multi-factorial similarity. These two dimensions are characterized by t-SNE1 and t-SNE2 in given graphs. γ_c_ expression was classified as ‘high’ or ‘low’ by an arbitrary threshold of top 10% or bottom 90% of γ_c_ mean fluorescence expressing CD4^+^ (Figures 1B,C) or CD4^+^/CD45RA_low_ (Figure 2) cells (for gating strategy see Supplementary Figure 1B).

For t-SNE analysis of a concatenated subgroup of CD4^+^ T-cells from healthy controls (*n* = 11) and T1D patients (*n* = 19) (Figure 1B) the following parameters were included: CD45RA and CCR7 (to identify naïve and memory T-cell subsets); IL-2Rα, IL-7Rα, IL-2Rβ, γ_c_ (to assess γ_c_ cytokine receptor expression and classify regulatory and effector T-cells). In a second step, t-SNE analysis was performed separately for CD45RA_low_ memory CD4^+^ T-cells from healthy controls (*n* = 20) and T1D patients (*n* = 25) (Figure 2A, left and right graph) and the following parameters were included: IL-2Rα, IL-7Rα, IL-2Rβ, γ_c_ (to assess γ_c_ cytokine receptor expression); CCR7 (to distinguish central memory and effector memory subpopulations).

### T-Cell Restimulation and Intracellular Cytokine Analysis

PBMC were immediately isolated by density gradient centrifugation with Biocoll (Biochrom AG) according to manufacturer's instructions. Without cryopreservation or batching, 2 × 10^5^ PBMC were cultured for a period of 14 h to harmonize experimental conditions between donors. This allowed sample processing on the next day independent of the respective bleeding time point and avoided suboptimal long *in vitro* restimulation for intracellular cytokine detection. Indirect effects of non-T cells during the pre-incubation period cannot be excluded, but since both groups were treated the same, a bias between T1D patients and controls is not likely. PBMC were then stimulated with 1μl/well human T-activator CD3/CD28 Dynabeads (Gibco) for 6 h. Golgi inhibitor Brefeldin A was added after 1 h. Thereafter cells were harvested and stained with Viability Dye dFluor e780 (eBioscience) following manufacturer‘s instructions. For intracellular cytokine analysis, cells were fixed and permeabilized using Cytokix/Cytoperm Buffer (BD Biosciences) according to manufacturer‘s instruction. Cells were stained using the following antibodies: CD4-BV510 (OKT4, Biolegend), CD8-BV785 (RPA-T8, Biolegend), CD45RA-FITC (HI100, Biolegend), CCR7-PeCy7 (3D12, BD), IFNγ-V450 (B27, BD), IL-21-PE (3A3-N2, Biolegend), TNFα-AF700 (Mab11, BD), and IL-2-PerCPCy5.5 (MQ1-17H12, Biolegend). Staining was performed in triplicates. Proportions of cytokine positive CD45RA_neg_ CD4^+^ memory T-cells were determined. Naïve CD4^+^ T-cells hardly produced cytokines after CD3/CD28 re-stimulation (data not shown). For data analysis FlowJo software (Miltenyi Biotech) was used. The gating procedure is depicted as Supplementary Figure 3A. Viability dye positive T-cells were excluded from further analyses. However, the proportion of these dead cells were low indicating no negative effects of the 14 h pre-incubation on cellular viability. Generally, T-cell proportions are calculated. Cytokine-producing T-cells in the present study were based on considerable T-cell numbers per well (median CD4^+^ T cell count: 9,577 cells) and CD3/28 induced cytokine-producing T-cell proportions ranged from 0.6% (median for IL-21) to 7.5% (median for IL-2). Therefore, cell numbers used for frequency calculations are sufficient to exclude overestimation of differences. The investigator was blinded to the group allocation when analyzing the data.

### STAT5 Phosphorylation

2 × 10^5^ PBMC were cultured for a period of 14 h. Afterwards, PBMC were stimulated with IL-2 (10IU/ml) or IL-7 (1ng/ml) for 15 min at 37°C and 5%CO_2_. Then, cells were fixed using true nuclear fixation buffer and permeabilized with methanol as described previously (37). Samples were then centrifuged, washed and stained with the following antibodies: CD4-AF700 (clone RPA-T4, Biolegend) and pSTAT5-PE (eBioscience). Stimulation and staining were performed in duplicates. For data analysis FlowJo software (Miltenyi Biotech) was used. After gating on CD4^+^ T-cells, the following gating procedure is depicted as Figure 4A. ΔpSTAT5 MFI was calculated as follows: MFI (pSTAT5 positive cells)—MFI (unstimulated cells). To assess STAT phosphorylation against the background of γ_c_ expression, study participants were stratified into “γ_c_ low” and a “γ_c_ high” subgroups according to γ_c_ expression on CD4^+^ T-cells: γ_c_ low (below the median γ_c_ MFI expression), γ_c_ high: (≥median γ_c_ MFI expression of all study participants).

### Measurement of Soluble γ_c_

Study participants serum was harvested and immediately stored at −80°C. After simultaneous thawing, soluble γ_c_ was measured using the Human Common gamma Chain/IL-2R gamma Duo Set ELISA kit (R&D) according to the manufacturer's instructions. All samples were analyzed in duplicates using an Infinite M200 ELISA reader (Tecan). Concentrations were calculated from respective standard curves on every plate by applying 4-parametric logistic regression. Samples outside the detection range were set to the corresponding lower (62.5 pg/ml) or upper range (8,000 pg/ml) value.

### Statistical Analysis

Graph Pad Prism 7 (Version 7.0a, GraphPad Software, La Jolla, CA) software was used for statistical analyses and figure preparation. Because of moderate study group sizes non-parametric distributions were assumed and statistical tests were chosen accordingly. The non-parametric Mann-Whitney U-test (two-tailed) was used to compare continuous characteristics of two study groups, the chi-squared test was used for categorical variables. For correlation analyzes, Spearman‘s correlation was used. *P*-values below 0.05 were considered statistically significant.

## Ethics Statement

This study was carried out in accordance with the recommendations of the Ethical Committee of the Medical Faculty of the Heinrich-Heine-University Duesseldorf, Germany (ID 4844) with written informed consent from all subjects. All subjects (older than 14 years) and their legal guardians gave written informed consent in accordance with the Declaration of Helsinki. The protocol was approved by the Ethical Committee of the Medical Faculty of the Heinrich-Heine-University Duesseldorf, Germany.

## Author Contributions

JS designed the study, performed experiments, analyzed and interpreted data, and wrote the manuscript. NM performed experiments, analyzed data, reviewed, and edited the manuscript. JAC, SK, and CR recruited patients and revised the manuscript. TM and EM contributed to the conceptualization and reviewed/edited the manuscript. MJ designed the study, analyzed and interpreted data, and wrote the manuscript. All of the contributing authors approved the final version of the manuscript. JS is the guarantor of this work, and as such, had full access to all the data in the study and takes responsibility for the integrity of the data and the accuracy of the data analysis.

### Conflict of Interest Statement

The authors declare that the research was conducted in the absence of any commercial or financial relationships that could be construed as a potential conflict of interest.
