# Supplementary material for: CD4+ T-Cells With High Common γ Chain Expression and Disturbed Cytokine Production Are Enriched in Children With Type-1 Diabetes
Source: Front Immunol. 2019 Apr 24;10:820. doi: 10.3389/fimmu.2019.00820 (PMC6499215; doi:10.3389/fimmu.2019.00820)
Supplement: Supplementary file 1 [file Presentation_1.PPTX]

## Slide 1
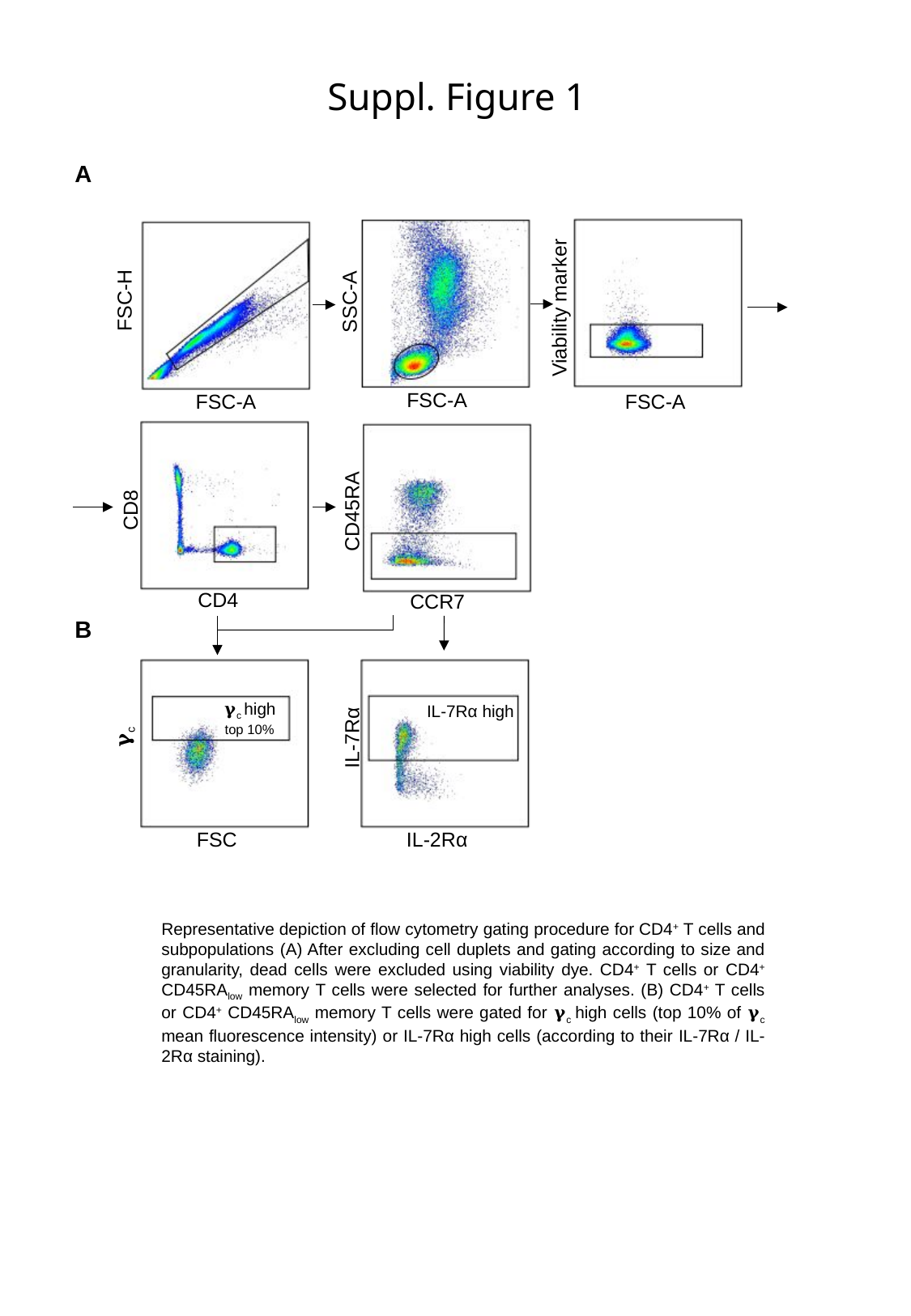

Suppl. Figure 1
A
FSC-H
SSC-A
Viability marker
FSC-A
FSC-A
FSC-A
CD8
CD45RA
CD4
CCR7
B
𝛄c high
top 10%
IL-7Rα high
𝛄c
IL-7Rα
FSC
IL-2Rα
Representative depiction of flow cytometry gating procedure for CD4+ T cells and subpopulations (A) After excluding cell duplets and gating according to size and granularity, dead cells were excluded using viability dye. CD4+ T cells or CD4+ CD45RAlow memory T cells were selected for further analyses. (B) CD4+ T cells or CD4+ CD45RAlow memory T cells were gated for 𝛄c high cells (top 10% of 𝛄c mean fluorescence intensity) or IL-7Rα high cells (according to their IL-7Rα / IL-2Rα staining).

## Slide 2
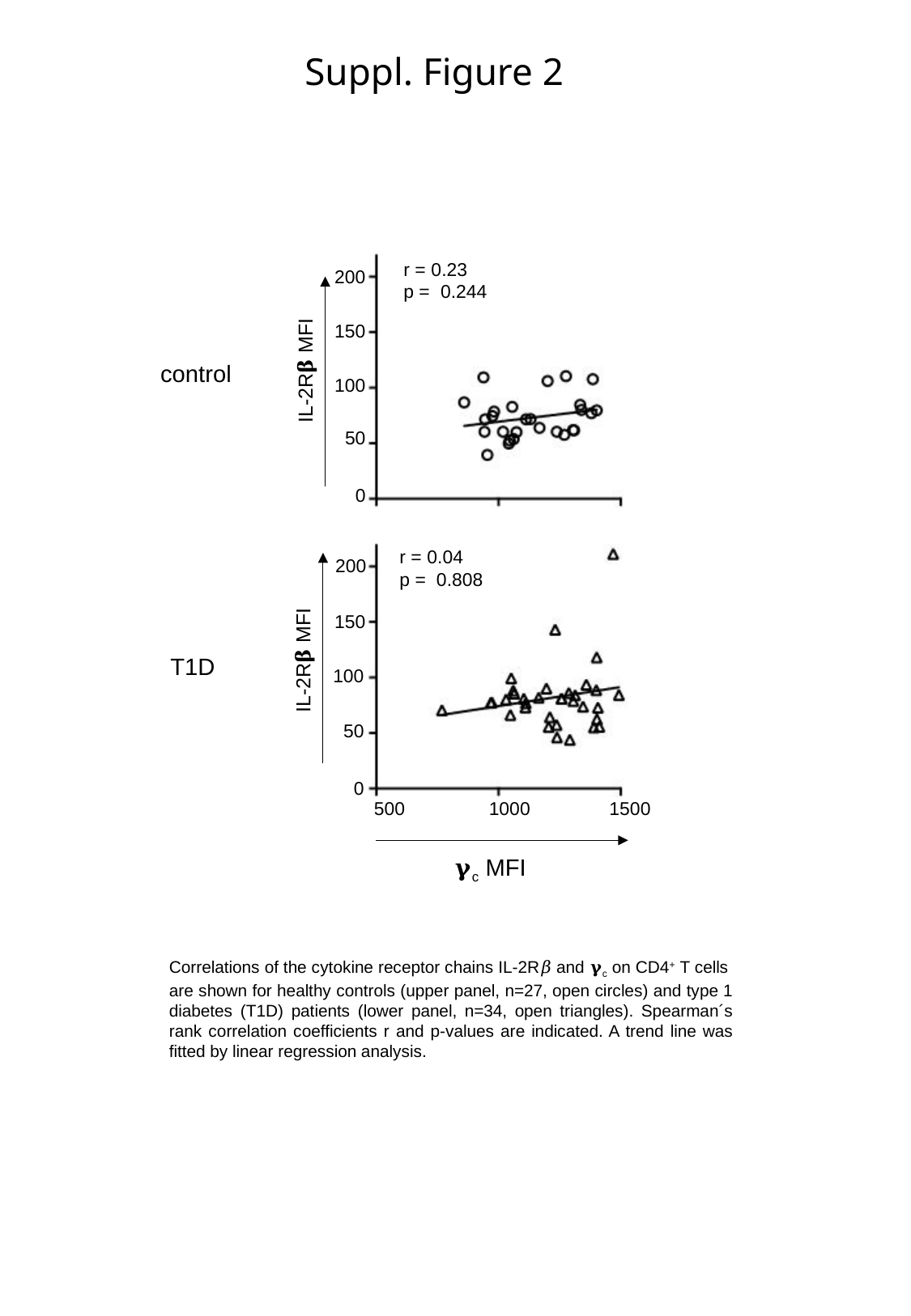

Suppl. Figure 2
r = 0.23
p = 0.244
200
150
IL-2R𝛃 MFI
control
100
50
0
r = 0.04
p = 0.808
200
150
IL-2R𝛃 MFI
T1D
100
50
0
500
1000
1500
𝛄c MFI
Correlations of the cytokine receptor chains IL-2R𝛽 and 𝛄c on CD4+ T cells are shown for healthy controls (upper panel, n=27, open circles) and type 1 diabetes (T1D) patients (lower panel, n=34, open triangles). Spearman´s rank correlation coefficients r and p-values are indicated. A trend line was fitted by linear regression analysis.

## Slide 3
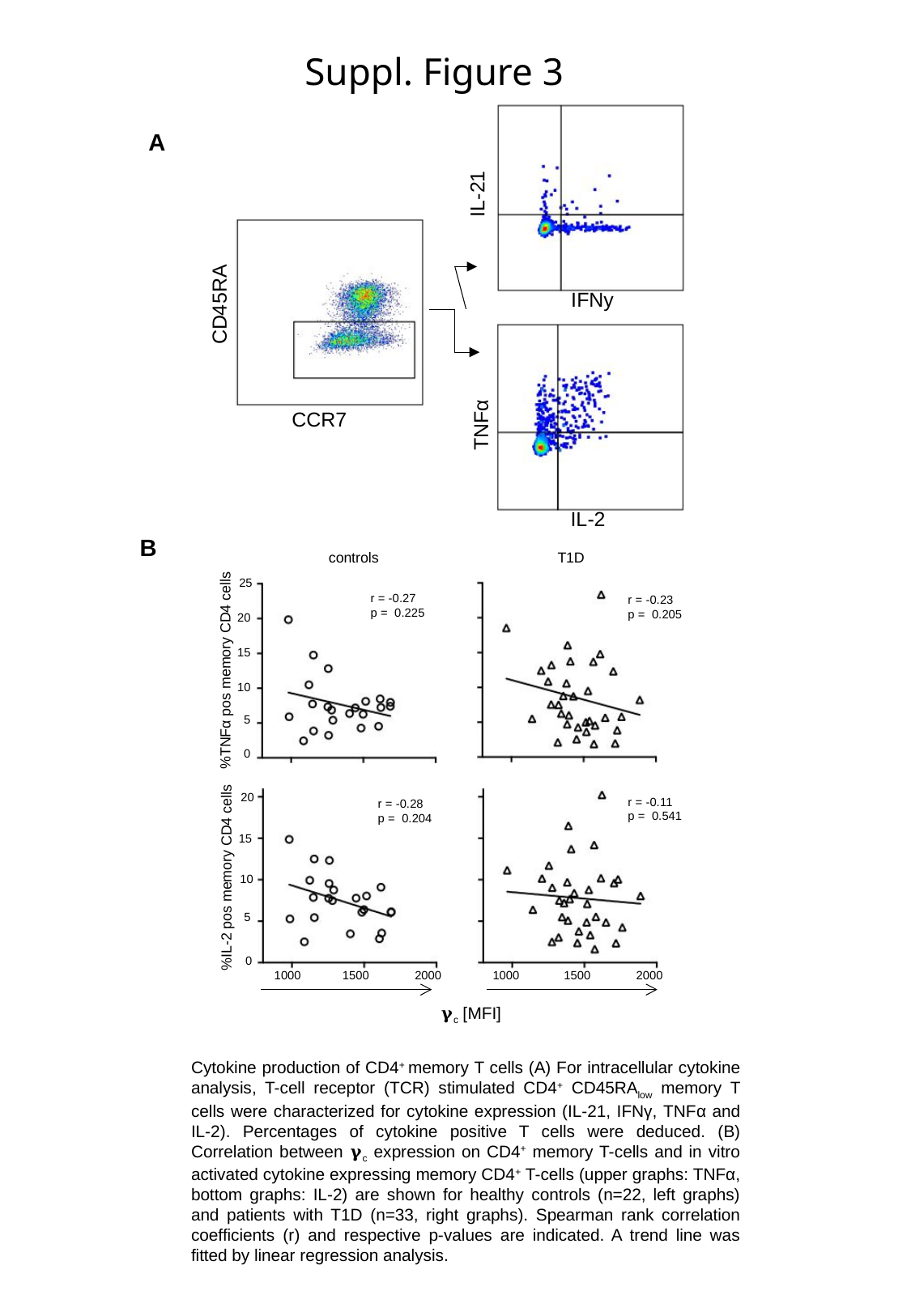

Suppl. Figure 3
IL-21
IFNy
CD45RA
CCR7
TNFα
IL-2
A
B
T1D
controls
25
r = -0.27
p = 0.225
r = -0.23
p = 0.205
20
15
%TNFα pos memory CD4 cells
10
5
0
20
r = -0.11
p = 0.541
r = -0.28
p = 0.204
15
%IL-2 pos memory CD4 cells
10
5
0
1000
1500
2000
1000
1500
2000
𝛄c [MFI]
Cytokine production of CD4+ memory T cells (A) For intracellular cytokine analysis, T-cell receptor (TCR) stimulated CD4+ CD45RAlow memory T cells were characterized for cytokine expression (IL-21, IFNγ, TNFα and IL-2). Percentages of cytokine positive T cells were deduced. (B) Correlation between 𝛄c expression on CD4+ memory T-cells and in vitro activated cytokine expressing memory CD4+ T-cells (upper graphs: TNFα, bottom graphs: IL-2) are shown for healthy controls (n=22, left graphs) and patients with T1D (n=33, right graphs). Spearman rank correlation coefficients (r) and respective p-values are indicated. A trend line was fitted by linear regression analysis.

## Slide 4
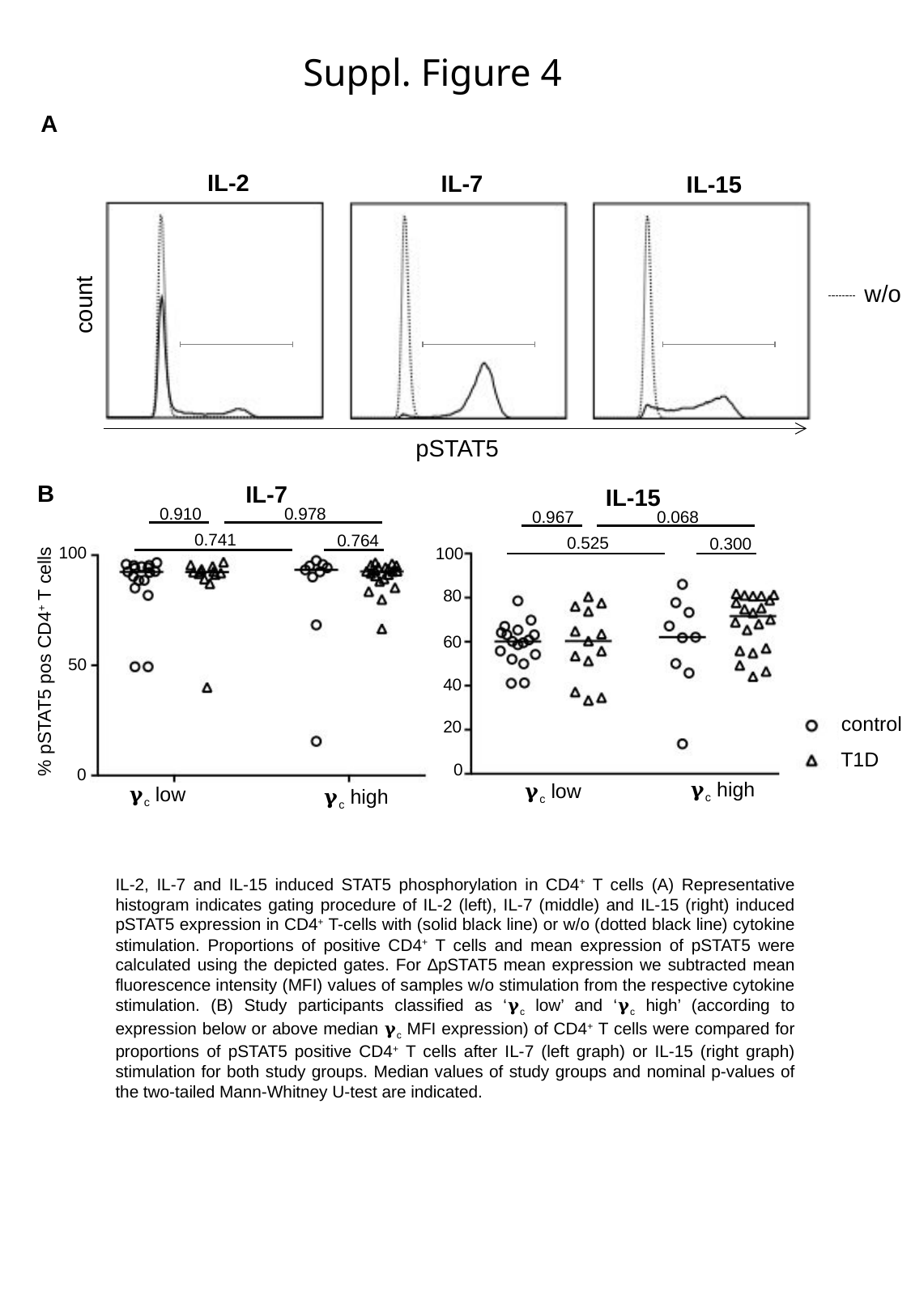

Suppl. Figure 4
A
IL-2
IL-7
IL-15
count
w/o
pSTAT5
B
IL-7
IL-15
0.978
0.910
0.741
0.764
0.068
0.967
0.525
0.300
100
100
80
60
% pSTAT5 pos CD4+ T cells
50
40
control
T1D
20
0
0
𝛄c high
𝛄c low
𝛄c low
𝛄c high
IL-2, IL-7 and IL-15 induced STAT5 phosphorylation in CD4+ T cells (A) Representative histogram indicates gating procedure of IL-2 (left), IL-7 (middle) and IL-15 (right) induced pSTAT5 expression in CD4+ T-cells with (solid black line) or w/o (dotted black line) cytokine stimulation. Proportions of positive CD4+ T cells and mean expression of pSTAT5 were calculated using the depicted gates. For ∆pSTAT5 mean expression we subtracted mean fluorescence intensity (MFI) values of samples w/o stimulation from the respective cytokine stimulation. (B) Study participants classified as ‘𝛄c low’ and ‘𝛄c high’ (according to expression below or above median 𝛄c MFI expression) of CD4+ T cells were compared for proportions of pSTAT5 positive CD4+ T cells after IL-7 (left graph) or IL-15 (right graph) stimulation for both study groups. Median values of study groups and nominal p-values of the two-tailed Mann-Whitney U-test are indicated.

## Slide 5
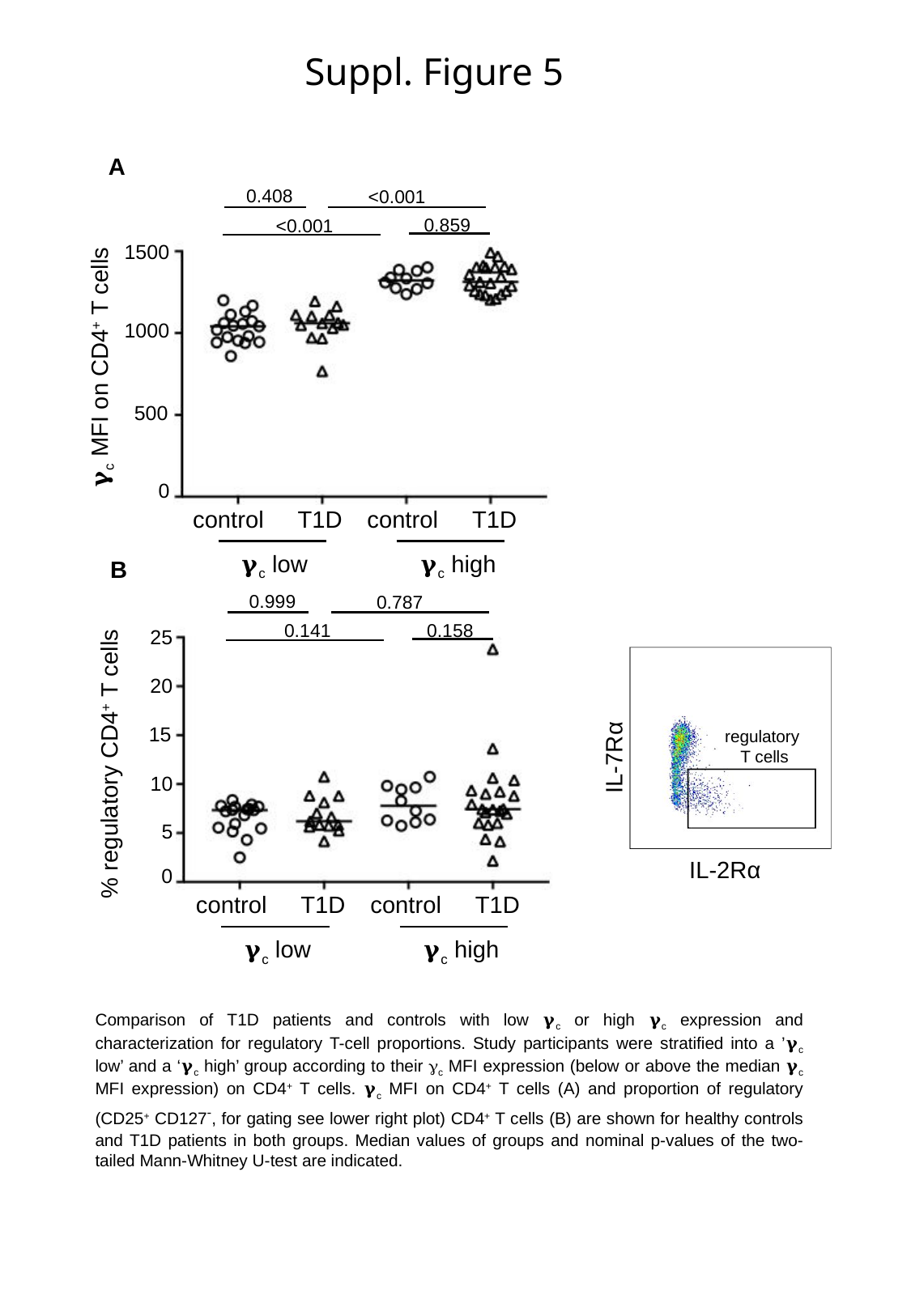

Suppl. Figure 5
A
0.408
<0.001
0.859
<0.001
1500
1000
𝛄c MFI on CD4+ T cells
500
0
control
T1D
control
T1D
𝛄c low
𝛄c high
B
0.999
0.787
0.158
0.141
25
20
15
% regulatory CD4+ T cells
10
5
0
control
T1D
control
T1D
𝛄c low
𝛄c high
regulatory
T cells
IL-7Rα
IL-2Rα
Comparison of T1D patients and controls with low 𝛄c or high 𝛄c expression and characterization for regulatory T-cell proportions. Study participants were stratified into a ’𝛄c low’ and a ‘𝛄c high’ group according to their γc MFI expression (below or above the median 𝛄c MFI expression) on CD4+ T cells. 𝛄c MFI on CD4+ T cells (A) and proportion of regulatory (CD25+ CD127-, for gating see lower right plot) CD4+ T cells (B) are shown for healthy controls and T1D patients in both groups. Median values of groups and nominal p-values of the two-tailed Mann-Whitney U-test are indicated.
